# Do inflammation and procoagulation biomarkers contribute to the metabolic syndrome cluster?

**DOI:** 10.1186/1743-7075-4-28

**Published:** 2007-12-21

**Authors:** Aldi T Kraja, Michael A Province, Donna Arnett, Lynne Wagenknecht, Weihong Tang, Paul N Hopkins, Luc Djoussé, Ingrid B Borecki

**Affiliations:** 1 Division of Statistical Genomics, Washington University School of Medicine, Saint Louis, MO, USA; 2 Department of Epidemiology, University of Alabama at Birmingham, Birmingham, AL, USA; 3 Department of Public Health Sciences, Wake Forest University School of Medicine, Winston-Salem, NC, USA; 4 Division of Epidemiology and Community Health, University of Minnesota, Minneapolis, MN, USA; 5 Department of Internal Medicine, University of Utah Health Sciences Center, Salt Lake City, UT, USA; 6 Department of Medicine, Brigham and Women's Hospital and Harvard Medical School, Boston, MA, USA

## Abstract

**Context:**

The metabolic syndrome (MetS), in addition to its lipid, metabolic, and anthropomorphic characteristics, is associated with a prothrombotic and the proinflammatory state. However, the relationship of inflammatory biomarkers to MetS is not clear.

**Objective:**

To study the association between a group of thrombotic and inflammatory biomarkers and the MetS.

**Methods:**

Ten conventional MetS risk variables and ten biomarkers were analyzed. Correlations, factor analysis, hexagonal binning, and regression of each biomarker with the National Cholesterol Education Program (NCEP) MetS categories were performed in the Family Heart Study (n = 2,762).

**Results:**

Subjects in the top 75% quartile for plasminogen activator inhibitor-1 (PAI1) had a 6.9 CI95 [4.2–11.2] greater odds (p < 0.0001) of being classified with the NCEP MetS. Significant associations of the corresponding top 75% quartile to MetS were identified for monocyte chemotactic protein 1 (MCP1, OR = 2.19), C-reactive protein (CRP, OR = 1.89), interleukin-6 (IL6, OR = 2.11), sICAM1 (OR = 1.61), and fibrinogen (OR = 1.86). PAI1 correlated significantly with all obesity and dyslipidemia variables. CRP had a high correlation with serum amyloid A (0.6) and IL6 (0.51), and a significant correlation with fibrinogen (0.46). Ten conventional quantitative risk factors were utilized to perform multivariate factor analysis. Individual inclusion, in this analysis of each biomarker, showed that, PAI1, CRP, IL6, and fibrinogen were the most important biomarkers that clustered with the MetS latent factors.

**Conclusion:**

PAI1 is an important risk factor for MetS. It correlates significantly with most of the variables studied, clusters in two latent factors related to obesity and lipids, and demonstrates the greatest relative odds of the 10 biomarkers studied with respect to the MetS. Three other biomarkers, CRP, IL6, and fibrinogen associate also importantly with the MetS cluster. These 4 biomarkers can contribute in the MetS risk assessment.

## Introduction

Metabolic syndrome (MetS) is a cluster of physiologic markers that includes obesity, insulin resistance, dyslipidemia, and hypertension. The National Cholesterol Education Program ATP III (NCEP) considers a proinflammatory and prothrombotic state as characteristic of metabolic syndrome (MetS) [1]. A group of biomarkers, products of proinflammatory and prothrombotic states, may play an important role in the cascade of the biochemical processes in the MetS development [2,3]. It has been suggested that imbalance in the energy associated with obesity, can drive all aspects of MetS, including the proinflammatory and prothrombotic states [3].

Inflammation and thrombosis are considered to influence the pathogenesis of coronary heart disease. Inflammation in general is associated with increased levels of leucocytes, fibrinogen, and C reactive protein (CRP) as well as other biomarkers. For example, Interleukin-6 (IL6) is considered an important inducer of the hepatic secretion of CRP. Plasma levels of IL6 and CRP have been reported to predict type 2 diabetes in humans [4]. It is assumed that the up-regulation of proinflammatory cytokines may trigger an impaired endothelial response and the production of other substances such as plasminogen activator inhibitor 1 (PAI1). The inflammation may serve as a stimulus to control procoagulation factors, downregulate procoagulants, and inhibit fibrinolysis [5-7].

Although several studies have used multivariate factor analysis as a tool for characterizing MetS clusters of risk variables [8-11], only a few have also included inflammatory markers [10,12-14]. Sakkinen *et al.*[12] studied 322 nondiabetic elderly. The traits were 11 conventional MetS risk variables and 10 procoagulation, inflammation and fybrinolysis biomarkers. They found that PAI1 clustered with the "body mass" latent factor while CRP, fibrinogen, DDimer, and a few other biomarkers clustered as an "inflammation" latent factor. PAI1 was also associated with the "insulin/glucose" factor. They hypothesized that obesity is related to impaired fibrinolysis. Meigs [9] commented on the failure of inflammatory markers to contribute substantially to the "body mass" factor, a surprising finding considering the fact that CRP was reported to be associated with BMI. For example, Laaksonen *et al.*[15] showed that middle-aged non-diabetic men with CRP levels ≥ 3 mg/l at baseline (with low grade inflammation), were 3 times more likely to develop MetS, but the risk weakened when they adjusted the data for BMI. Similar findings for three ethnicities were described by Hanley *et al.*[14] (n = 1,087). They found PAI1 contributed to a "metabolic syndrome" factor, whereas fibrinogen and CRP contributed to an "inflammation" factor. Also, Yudkin *et al.*[10] (n = 393) reported that fibrinogen, CRP, and IL6 contributed together in a separate "inflammation" factor, but with a very low contribution in the "metabolic syndrome" factor. Recently, Tang *et al.*[13] utilized two biomarkers as part of the risk factors for MetS. Studying data in the Family Heart Study, they found factor 1 as the most representative of MetS where PAI1 was an important contributor.

We studied ten conventional MetS risk variables in the NHLBI Family Heart Study (Time 2, see **Methods**). Their combinations in a multivariate factor analysis generated factors of MetS. A subgroup of the conventional variables in our study represented the set of the variables utilized to qualitatively characterize MetS (NCEP definition, see **Methods**). Consequently, the 10 conventional variables served as a representative construct for MetS. Adding ten prothrombotic and proinflammatory biomarkers in the study provided an ample vision to assess the role of ten biomarkers in the MetS cluster.

## Methods

### Sampled population

The NHLBI Family Heart Study (FHS-Time 2) is a family-based study that investigates genetic and non-genetic causes of coronary calcification, atherosclerosis, and cardiovascular risk factors during a 2^nd ^visit of selected FHS families. The white cohort (n = 2,762, including 19 participants with race undefined or "other") was recruited through 2002–2003 at five centers. Details of this study are reported elsewhere [16,17]. The exact numbers of subjects analyzed varied, because some of the biomarker assays were available only for a smaller group of participants (see the corresponding tables and figures for the exact sample sizes).

### Risk variables

Ten MetS conventional risk variables were studied: body mass index (BMI, kg/m^2^), waist circumference (WAIST, cm), waist to hip ratio (WHR), percent body fat (PBF), fasting (≥ 12 hours) glucose (GLUC, mg/dl), high density lipoprotein cholesterol (HDLC, mg/dl), tryglicerides (TG, mg/dl), low density lipoprotein cholesterol (LDLC, mg/dl), and systolic (SBP, mm Hg) and diastolic blood pressure (DBP, mm Hg). PBF was measured as the bioelectric impedance of the body and was calculated based on the Lukaski formula [18]. The venipuncture blood samples were drawn and collected at each field center. Blood biochemistries were quantified at the Central Biochemistry Laboratory at Fairview-University Medical Center in Minneapolis, MN, described in detail elsewhere [17].

Ten inflammatory, prothrombotic, or fibrinolysis biomarkers were tested for their contribution to the MetS, respectively CRP, fibrin fragment D-Dimer (DDIMER), fibrinogen, interleukin-2 soluble receptor alpha (IL2SR), IL6, monocyte chemotactic protein 1 (MCP1), matrix metalloproteinase (MMP3), PAI1, serum amyloid A (SAA), and soluble intercellular adhesion molecule 1 (sICAM1) (Table 1). Eight of the biochemical assays on the inflammatory markers were performed at the Laboratory for Clinical Biochemistry Research, University of Vermont, Colchester, VT. PAI1 and fibrinogen assays were performed at the Central Biochemistry Laboratory at Fairview-University Medical Center in Minneapolis, MN. The following assays were carried out:

**Table 1:** Participants' characteristics

**Variables**		**Mean**	**Std.Dev.**
**FHS-T2 Whites**	**(n = 2762)**		
Females (%)	**55**		
AGE	2762	**57**	**13**
BMI	2761	**28.9**	**5.7**
WAIST	2758	**98.9**	**16.3**
WHR	2758	**0.9**	**0.1**
PBF	2385	**33.1**	**9.4**
GLUC	2362	**99.8**	**21.7**
HDLC	2747	**48.8**	**14.4**
LDLC	2694	**110.6**	**33.2**
TG	2749	**144.3**	**93.7**
SBP	2760	**121**	**20**
DBP	2760	**70**	**10**
CRP	2685	**3.6**	**5.7**
SICAM1	2726	**247.8**	**79.0**
MCP1	2615	**196.6**	**91.7**
**FHS-T2 Whites subsample**	**(n = 532)**		
FIBRINOGEN	415	**284.9**	**62.1**
PAI1	415	**39.6**	**44.4**
IL2SR	474	**802.0**	**457.8**
IL6	520	**2.4**	**1.6**
MMP3	525	**16.9**	**11.1**
SAA	516	**6.8**	**18.8**
DDIMER	532	**0.4**	**0.4**

Note: For the measurement units and the full name of each variable, see paragraph "**Risk variables**" in the **Methods**.

**CRP **(mg/l) was measured using the BNII nephelometer from Dade Behring utilizing a particle enhanced immunonepholometric assay. Intra-assay coefficient of variation (IACV) ranged from 2.3 – 4.4% and inter-assay coefficient of variation (IRCV) ranged from 2.1 – 5.7%.

**DDIMER **(ug/ml) was measured using an immuno-turbidimetric assay (Liatest D-DI, Stago) on the Sta-R analyzer (Stago) [19].

**Fibrinogen **(mg/dl) concentrations were quantified by the STAR automated coagulation analyzer (Diagnostica Stago), and the clotting method of Clauss [20]. In this method, the level of fibrinogen is directly correlated to the clotting time of a diluted plasma sample in the presence of excess thrombin. Participants' fibrinogen results were then validated based on control results. The IACV was 4% and the IRCV was 4%.

**IL2SR **(pg/ml) was measured by ultra-sensitive ELISA (R&D Systems, Minneapolis, MN) [21]. IACV was 3.0% and IRCV was 5.0%.

**IL6 **(pg/ml) a pro-inflammatory cytokine was measured by ultra-sensitive ELISA (R&D Systems, Minneapolis, MN) [22]. The IRCV was about 6.3%.

**MCP1 **(pg/ml) was measured using an ultra-sensitive ELISA assay (Quantikine Human MCP-1 Immunoassay; R&D Systems, Minneapolis, MN). The sample type used was citrated plasma. The IACV and IRCV ranged 4.7–7.8% and 4.6–6.7% respectively.

**MMP3 **(ng/ml) was measured by an ultra-sensitive, solid-phase sandwich ELISA using a polyclonal antibody specific for both the pro- and active forms of MMP-3 (Quantikine Human MMP-3 (total) Immunoassay; R&D Systems, Minneapolis, MN). The IACV and IRCV ranged from 5.7–6.4% and 7.0–8.6%, respectively.

**PAI1 **(ng/ml) assay is sensitive to free PAI1 (both latent and active), but not PAI1 in complex with t-PA. It is done as a two-site ELISA. The analytical CV for this assay was 3.47% [23-25].

**SAA **(mg/l) was measured with the BNII nephelometer from Dade Behring utilizing a particle enhanced immunonepholometric assay (N Latex SAA; Dade Behring Inc., Deerfield, IL). IACV ranged from 4.3–6.2% and IACV ranged from 2.8–4.7%.

**sICAM1 **(ng/ml) was measured by an ELISA assay (Parameter Human SICAM-1 Immunoassay; R&D Systems, Minneapolis, MN). The laboratory CV was 5.0% [26,27].

### Metabolic syndrome

A grouping of at least any 3 of the 5 following **MetS categories** beyond the NCEP defined thresholds in a subject, was considered as a clinical expression of the NCEP MetS: Abdominal obesity, given as *large WAIST *for men >102 cm and > 88 cm for women; *high fasting GLUC *≥ 110 mg/dl or medication for diabetes; *high TG *≥ 150 mg/dl; *low HDLC *< 40 mg/dl in men or < 50 mg/dl in women; *high blood pressure *(SBP/DBP) ≥ 130/≥ 85 mm Hg or antihypertensive medication use [1].

### Statistical analysis

Variables of interest were expressed as mean ± SD (Table 1). In addition, four statistical techniques were applied to investigate the contribution of 10 biomarkers onto the conventional MetS risk variables clusters and/or to the MetS categories.

First, the multivariate factor analysis was performed with the *FACTANAL *function, with the maximum likelihood estimate option and the "Varimax" rotation, in Splus version 6.2 of Insightful Corp., Seattle, WA. This method was performed first on 10 conventional MetS risk variables. After, one biomarker was added in the analysis, subject to the sample sizes available, to discover any cluster of each biomarker onto the identified latent factors. The acceptance of an extra factor in the model was made by applying two general rules, the p-value of the model had to be < 0.05, and the last factor had to have at least two contributing risk variables (with a contributing coefficient about 0.4 or above). We stopped beyond the 5-th factor because the additional factors represented only single variables. The contributing coefficients for the standardized variables correspond to correlation coefficients of the original risk variables to the latent factors. A contributing coefficient value in the tables greater/equal to 0.4 was highlighted in bold and is considered as having a substantial influence on the factor. In advance to factor analysis, distributions of all risk variables were checked for their fit to a normal distribution. Variables BMI, WHR, HDLC, TG, CRP, DDIMER, fibrinogen, IL2SR, IL6, MCP1, MMP3, PAI1, SAA, and SICAM1, were natural log transformed. GLUC was expressed as the inverse of a power transformation (1/GLUC^2^). Risk variables went also through covariate adjustments, in a stepwise regression in SAS v. 9.1.3 for Linux OS, for age, age^2^, age^3 ^and recruitment center, within gender. The residuals were standardized to mean 0 and variance 1.

Second, Pearson correlations were produced on the above preprocessed data, by using the PROC CORR of SAS (Table 2).

**Table 2:** Correlation matrix of 20 risk variables in the FHS-T2 Whites

**FHS-T2**	**BMI**	**PBF**	**WAIST**	**WHR**	**GLUC**	**LDLC**	**HDLC**	**TG**	**SBP**	**DBP**	**CRP**	**SICAM1**	**FIB**	**PAI1**	**IL2SR**	**IL6**	**MCP1**	**MMP3**	**SAA**
**PBF**	**0.73‡**		**-**	**-**	**-**	**-**	**-**	**-**	**-**	**-**	**-**	**-**	**-**	**-**	**-**	**-**	**-**	**-**	**-**
**WAIST**	**0.89‡**	**0.70‡**		**-**	**-**	**-**	**-**	**-**	**-**	**-**	**-**	**-**	**-**	**-**	**-**	**-**	**-**	**-**	**-**
**WHR**	**0.52‡**	**0.42‡**	**0.74‡**		**-**	**-**	**-**	**-**	**-**	**-**	**-**	**-**	**-**	**-**	**-**	**-**	**-**	**-**	**-**
**GLUC**	**-0.33‡**	**-0.24‡**	**-0.32‡**	**-0.27‡**		**-**	**-**	**-**	**-**	**-**	**-**	**-**	**-**	**-**	**-**	**-**	**-**	**-**	**-**
**LDLC**	0.01	0.07†	0.00	0.02	0.08†		**-**	**-**	**-**	**-**	**-**	**-**	**-**	**-**	**-**	**-**	**-**	**-**	**-**
**HDLC**	**-0.28‡**	**-0.22‡**	**-0.26‡**	**-0.22‡**	**0.17‡**	-0.02		**-**	**-**	**-**	**-**	**-**	**-**	**-**	**-**	**-**	**-**	**-**	**-**
**TG**	**0.29‡**	**0.27‡**	**0.29‡**	**0.28‡**	**-0.17‡**	**0.09‡**	**-0.46‡**		**-**	**-**	**-**	**-**	**-**	**-**	**-**	**-**	**-**	**-**	**-**
**SBP**	**0.19‡**	**0.17‡**	**0.18‡**	**0.14‡**	**-0.14‡**	0.07†	0.00	**0.13‡**		**-**	**-**	**-**	**-**	**-**	**-**	**-**	**-**	**-**	**-**
**DBP**	0.05*	0.04	**0.06†**	**0.08‡**	-0.03	**0.11‡**	0.04*	**0.09‡**	**0.69‡**		**-**	**-**	**-**	**-**	**-**	**-**	**-**	**-**	**-**
**CRP**	**0.35‡**	**0.35‡**	**0.37‡**	**0.28‡**	**-0.15‡**	0.02	**-0.13‡**	**0.20‡**	**0.15‡**	0.06†		**-**	**-**	**-**	**-**	**-**	**-**	**-**	**-**
**SICAM1**	**0.08‡**	**0.08‡**	**0.09‡**	**0.11‡**	**-0.08‡**	-0.05†	**-0.15‡**	**0.11‡**	0.02	0.00	**0.15‡**		**-**	**-**	**-**	**-**	**-**	**-**	**-**
**FIB**	**0.28‡**	**0.32‡**	**0.32‡**	**0.33‡**	-0.13*	0.06	**-0.19‡**	0.11*	0.14†	0.00	**0.47‡**	0.12*		**-**	**-**	**-**	**-**	**-**	**-**
**PAI1**	**0.53‡**	**0.51‡**	**0.56‡**	**0.43‡**	**-0.37‡**	0.10*	**-0.39‡**	**0.43‡**	**0.25‡**	**0.17‡**	**0.28‡**	**0.19‡**	0.16†		**-**	**-**	**-**	**-**	**-**
**IL2SR**	0.04	0.01	0.10*	0.11*	-0.04	-0.10*	-0.17†	0.08	0.03	-0.08	**0.28‡**	**0.33‡**	0.37*	0.02		**-**	**-**	**-**	**-**
**IL6**	**0.37‡**	**0.34‡**	**0.39‡**	**0.30‡**	**-0.30‡**	0.00	**-0.23‡**	**0.20‡**	**0.16‡**	0.02	**0.51‡**	**0.28‡**	0.12	0.49†	**0.32‡**		**-**	**-**	**-**
**MCP1**	**-0.14‡**	**-0.16‡**	**-0.16‡**	**-0.15‡**	**0.08‡**	-0.03	**0.19‡**	**-0.20‡**	-0.05*	0.00	**-0.08‡**	**-0.16‡**	-0.09	**-0.26‡**	**-0.23‡**	**-0.30‡**		**-**	**-**
**MMP3**	-0.06	-0.08	-0.07	-0.07	0.05	-0.08	0.02	-0.09*	0.04	0.00	-0.05	0.02	0.26	-0.18	0.12†	0.06	0.01		**-**
**SAA**	**0.20‡**	0.16†	**0.19‡**	**0.15‡**	-0.11*	0.02	-0.01	0.10*	0.13†	0.07	**0.60‡**	0.15†	-0.28	0.06	**0.21‡**	**0.38‡**	-0.06	0.06	
**DDIMER**	0.01	0.01	0.03	0.05	0.02	-0.05	-0.01	0.00	0.02	-0.01	0.17†	0.04	0.2	0.12†	0.13†	**0.16‡**	-0.10*	0.12†	0.11*

*p < .05;†p < .01; **‡ p < .0001**; Negative sign of GLUC correlations is result of GLUC transformation (see Methods)

Third, a binary association between the PRESENCE/ABSENCE of a participant in the top 75% quartile, as well as in the 50% of the data for each biomarker and if CLASSIFIED/NOT with MetS was evaluated through logistic regression (PROC LOGISTIC of SAS).

In addition, a graphical technique named "hexagonal binning" was implemented [28]. For its methodology, results, and discussion see the **Appendix**.

## Results

**Figure 1:**
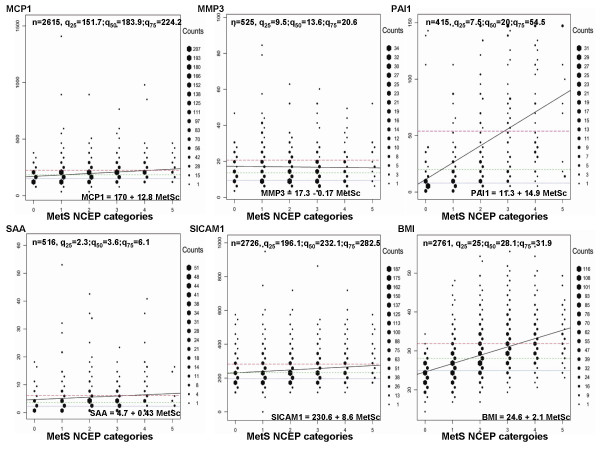
Hexagonal binning of MCP1, MMP3, PAI1, SAA, and sICAM1 against NCEP MetS categories (0–5). BMI was added in the figure only for comparison. At each sub-graph the following information is provided: *top of the graph*: **n **– the sample size studied for the particular biomarker; **q**_25 _– the raw value of biomarker at the 25th percentile, **q**_50 _– the 50th percentile, and **q**_75 _– the 75th percentile; *bottom of the graph*: **regression equation **of the fitted model for a biomarker on MetS NCEP categories; *NCEP MetS categories*: 0 – none of the 5 categories reached beyond the NCEP thresholds (see **Metabolic syndrome **paragraph in the **Methods **section); 1 – at least one of the 5 categories passed the NCEP thresholds; similarly, 5 – all 5 categories passed the MetS NCEP thresholds. Three horizontal dashed lines represented the 25% (gray color), 50% (green color), and the 75% (red color) quartiles of a biomarker distribution. (Details provided in the **Results **and in the **Appendix**). For presentation clarity, 1 data point above 2500 pg/ml for MCP1, and 3 data points above 100 mg/l for SAA were removed from the graph.

**Figure 2:**
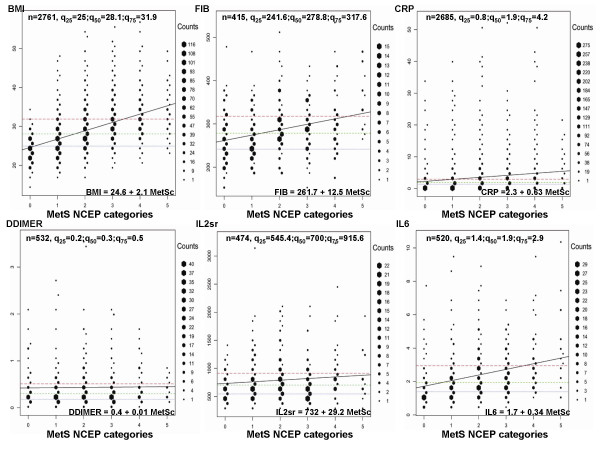
Hexagonal binning of fibrinogen (FIB), CRP, DDIMER, IL2SR, and IL6 against NCEP MetS categories (0–5). BMI was added in the figure only for comparison. (Details provided in the **Results **and in the **Appendix**). For presentation clarity 2 data points above 70 mg/l for CRP, and 1 data point above 6000 pg/ml for IL2SR were removed from the graph.

Participants between ages 30 and 93 had a mean BMI of 28.9 ± 5.68 (Table 1). PAI1 levels were relatively high (39.58 ± 44.36) with an interquartile range (IR) of 46 between the 25% quartile (q_25_) and 75% quartile (q_75_) (see Table 1 and Figure 1). The CRP mean level was 3.61 ± 5.65. The distribution is shown in Figure 2 which indicates an IR of CRP of 3.4 in a sample of 2,685 participants. The central 50% of the data were more spread around the mean for PAI1 than for CRP. Using the ratio of IR/SD we found that fibrinogen, sICAM1, PAI1, IL6, and MMP3 compared to BMI, had a similar spread of the central 50% of the data. In contrast, the central 50% of the data were less spread around the mean compared to BMI for SAA, CRP, DDIMER, IL2sr, and MCP1.

It is interesting to note that PAI1 correlated significantly with all obesity variables (with BMI, 0.53; PBF, 0.51; WAIST, 0.56), IL6 (0.49), dyslipidemia, even with SBP and DBP, reaching almost the lowest but still significant correlation with fibrinogen (Table 2). CRP had high correlations with SAA, IL6, and fibrinogen. On the other hand, correlations of sICAM1, MCP1, MMP3, and DDIMER, did not pass a coefficient of 0.4 with any of the 19 other risk variables and had a general trend for lower correlations (Table 2 and Figure 2).

The above correlation matrix reflected in the results of factor analysis. The factor analysis with "Varimax" rotation on the 10 conventional risk variables produced 4 latent factors. The first factor was a contribution of BMI, WAIST, PBF, and more modest contributions of WHR and GLUC. The second factor was mostly SBP and DBP contributions. The third factor was contributed by TG, HDLC, with less contribution of GLUC, LDLC, and PBF. The last factor represented a contribution of WHR and WAIST. The MetS cluster of latent factors explained about 60% of the total variance of the conventional risk variables.

**Figure 3:**
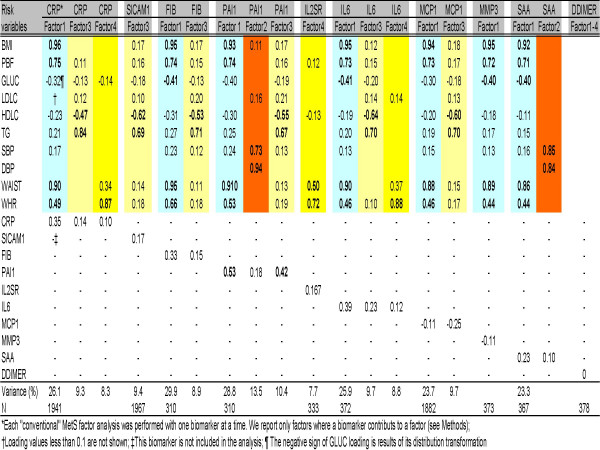
Factor analysis of "conventional" MetS risk factors with the addition of an individual biomarker at a time. Colors coded are factor domains: blue – Factor 1 – obesity; orange – Factor 2 – blood pressure; yellow – Factor 3 – lipids; and gold – Factor 4 – central obesity. Reported are only factors where a biomarker contributed.

Figure 3 is a compilation of different factor analyses. Each biomarker was individually added in the factor analysis of the MetS cluster. PAI1 had the strongest contribution compared to other biomarkers (loading coefficients 0.53 in Factor 1 and 0.42 in Factor 3). CRP contributed mainly in Factor 1. IL6 was quite similar in contribution as CRP. When CRP and fibrinogen were combined in the MetS factor analysis (data not shown) only in that case, CRP and fibrinogen stood in a separate factor, and loading coefficients were 0.8 for CRP and 0.56 for fibrinogen. sICAM1 contributed in the lipid factor (Factor 3). The rest of the biomarkers, DDIMER, IL2SR, MCP1, MMP3, and SAA had a limited contribution to the factor analysis of MetS cluster.

**Figure 4:**
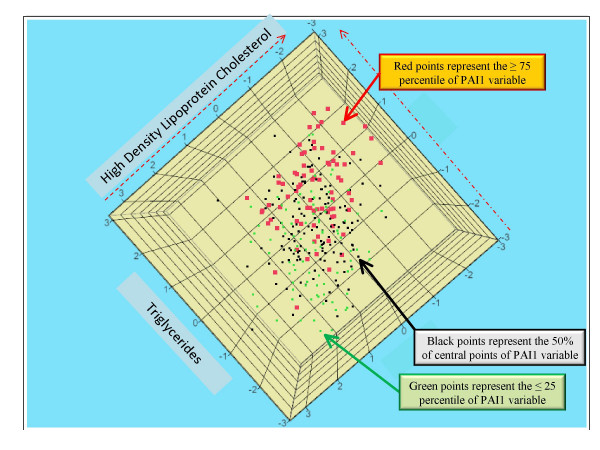
A three-dimensional presentation of relations among PAI1, HDLC, and TG, which accounts for only a small number of relationships of PAI1 with the classical risk factors of MetS. The three variables were natural log transformed and adjusted for a few covariates (for details see **Statistical analysis **paragraph in the **Methods **section). Red points represent the upper 25th percentile based on PAI1 values. Green points represent the lower 25th percentile, and in black are the 50% central PAI1 values. The higher TG values in a subject were associated with a lower HDLC value and also with a trend of increased values of PAI1.

Figure 4 shows only a partial picture of the PAI1 relationships to classical MetS risk factors, in particular to HDLC and TG. Three variables presented in this figure, HDLC, TG, and PAI1 represent standardized residuals after natural log transformation and adjustments (for details see the paragraph of **Statistical analysis **in the **Methods **section). A red color annotates PAI1 values above the 75th PAI1 percentile; a green color annotates PAI1 values below the 25th percentile; and a black color annotates the 50% central data of PAI1 distribution. The locations of these points in Figure 4 are dependent on the HDLC and TG values of the subjects. It is clear that TG values along with PAI1 values increased while HDLC values decreased. This is in distinct contrast with LDLC, which had little to no significant correlations with most of the biomarkers studied.

The other graphical technique employed by us to better understand relationships of MetS NCEP categories and biomarkers is hexagonal binning. This technique helps visually to better investigate the predictive evidence of biomarkers to the NCEP MetS categories (see details in the **Appendix**, and Figures 1 and 2). More than 25% of participants were obese (with a BMI of 30 kg/m^2 ^or greater). Of them, from the BMI top 75% percentile only a subset, 16.3% classified with MetS. Another 18% of the data, located in the BMI central 50%, classified with MetS. PAI1 showed a similar pattern as BMI. PAI1 fitted regression line intersected with the 75% quartile line at the graphical point of 3 MetS categories, confining 17.1% of the sample with MetS, and an additional 15.7% with MetS between q_25 _and q_75 _lines. Differently, the CRP fitted regression line intersected with the CRP top 75% quartile line at the graphical point of 1 MetS NCEP category. At CRP values 4.2 mg/l or greater, 11.8% of the sample were classified with MetS. A subsample (20.7%) classified with MetS was represented by participants having CRP values between q_25 _and q_75 _lines. IL6 and fibrinogen respectively confined 13.2% and 11.6% of the sample, with MetS and placed at the top biomarkers' 75% percentiles. In contrast, DDIMER and MMP3 had flat regression lines.

PAI1 had the highest odds (6.9, CI95 [4.2–11.2], p < 0.0001) and comparable with BMI odds (5.2, [4.3–6.2]) when participants were in the top 75% quartile and classified as having the NCEP MetS. Other biomarkers MCP1 (OR = 2.19, CI95 [1.8–2.6], p < 0.0001), CRP (OR = 1.89, [1.6–2.3], p < 0.0001), IL6 (OR = 2.11, [1.4–3.1], p = 0.0002), sICAM1 (OR = 1.61, [1.4–1.9], p < 0.0001), and fibrinogen (OR = 1.86, [1.2–2.9], p = 0.0066) had also significant odds for participants being in the top 75% quartile of the biomarker distribution and classified as having the NCEP MetS. The association of biomarker values per participant within the corresponding central 50% of the data and being classified with MetS, was significant only for CRP (OR = 1.3, [1.1–1.5], p = 0.0007).

## Discussion

Inflammation, procoagulation and fibrinolysis biomarkers have been recently hypothesized to be associated with MetS [12] and the development of atherosclerosis [29]. In our study, PAI1 represented an important contributor to the obesity and lipids factors and associated with the NCEP MetS. Similar to our findings, PAI1 had a significant contribution in the "Metabolic syndrome" factor in the Hanly *et al.*[14] and in the Tang *et al.*[13], and in the "Body mass" and lesser in the "Insulin/glucose" factors in Sakkinen et al. studies [12]. PAI1 was strongly associated with MetS components such as BMI, TG, a homeostasis model assessment of insulin resistance, hs-CRP, and alanine aminotransferase [30]. PAI1 prevents plasmin generation and is considered a primary inhibitor of fibrinolysis [31], and of extracellular matrix degradation [32]. Similar to CRP, PAI1 is lowered by weight loss and drugs that improve insulin sensitivity. PAI deficient mice were resistant to diet induced obesity, explained by increased energy expenditure [33]. PAI1 is induced by many proinflammatory and pro-oxidant factors. For example, TNFα (tumor necrosis factor alpha) is an inducer of the increased levels of PAI1 [34-37]. Transgenic mice with elevated levels of PAI1 in plasma developed venous occlusions [38]. Transgenic mice with overexpression of human PAI1 were found to develop macrovascular coronary thrombosis and subendocardial myocardial infarction [39]. Smith *et al.*[40] concluded that PAI1 together with fibrinogen, DDIMER were associated significantly with risk of CVD. Consequently, PAI1 has important functions and is a biomarker that can increase the prediction for MetS in addition to the conventional risk factors. Mertens *et al.*[7] investigated the association of 5 biomarkers with the MetS as defined by the NCEP criteria in 520 overweight and obese subjects, concluded that PAI1 is a true component of MetS. However, questions remain open whether PAI1 genes contribute to the MetS development or represent a step in the cascade of the biochemical pathways of MetS. It has been proposed that as a result of obesity and hypoxia white adipose tissue overspills inflammation markers [41]. Skurk and Hauner [42] reviewed obesity and impaired fibrinolysis and reported that increased fat cell size and adipose tissue mass associate with higher offerings of PAI1 in circulation. Moreover, Liang *et al.*[33] studying cultured adipocytes from PAI1 (+/+) and PAI1(-/-) mice, found that PAI1 deficiency had a protective role against insulin resistance, and was also associated with up- and down-regulation of some other important genes.

A large number of studies have scrutinized the role of CRP. Bassuk *et al.*[4], Ridker *et al.*[43], and Biasucci *et al.*[44] summarized that CRP predicts incident myocardial infraction, stroke, peripheral arterial disease, sudden cardiac death, and confers additional prognostic value at all levels of MetS. In contrast, Lloyd-Jones and Greenland [45] argued that there is no advantage in adding CRP to the standard risk model for coronary vascular disease (CVD). The CRP predictive values were also explored by Pankow *et al.*[46] who found that BMI, WHR and the prevalent diabetes together explained 30% and 22% of inter-individual variability in the CRP levels respectively in women and men. In our study CRP contributes a small fraction to the obesity factor (a loading of 0.35) and less to the lipids factors (a loading of 0.14). Quite the opposite, CRP clustered well in an independent latent factor with fibrinogen (a loading of 0.8, data not shown). The trends in our correlation findings for CRP matched with the ones found in other publications. For example, CRP had a high correlation 0.47 (Sakkinen *et al.*[12]) and 0.5 (Hanley *et al.*[14]) with fibrinogen, as two markers of the acute-phase response.

Another interesting biomarker with significant findings in our data was IL6. On severe exercise, IL6 is released in large quantities from the skeletal muscle [47]. IL6 has been suggested to be involved in the insulin sensitivity in mice [48,49], and recently it is suggested that IL6 might influence glucose tolerance [50,51]. Elevated levels of IL6 are found to be a predictor of CVD [52]. In our data, IL6 contributed to obesity and less to the lipids factor. A unique finding was a high correlation between IL6 with CRP, and PAI1, and lower although significant with BMI, GLUC, WAIST, WHR, and SAA.

sICAM1 is an important adhesion molecule and is implicated to mediate the firm adhesion of leukocytes into endothelium [53]. sICAM1 in our data correlated positively and significantly with PAI1 and CRP. Other studied biomarkers such as MCP1, SAA, and IL2SR had significant correlations with CRP. Of them, SAA is an acute-phase protein that exhibits a strong rise in blood in response to inflammation. In our data the SAA response to inflammation was depicted with its highest significant correlation (0.6) to CRP, but was not associated with MetS.

It has been hypothesized that a chronic, mild inflammatory state is the ground of MetS. Besides, Dancan and Schmidt [54] suggested that chronic activation of the innate immune system can be related to MetS. Recently, Forsythe *et al.*[55] showed that low carbohydrate diet decreased greatly TNF-α, IL6, IL8, MCP1, ICAM, and PAI1. Based on these and several other reported facts, we speculate that some of these biomarkers are not only biochemical steps in the cascades of inflammation, fibrinolysis and prothrombosis, but also markers of obesity and dyslipidemia. DDIMER and MMP3 in our data could not be used as direct predictors of the NCEP MetS, while PAI1, CRP, IL6, and fibrinogen can convey additional information on individuals classified with MetS. In particular PAI1, a key component in the biochemical cascade of fibrinolysis, may simultaneously influence fibrinolysis and coagulation which may contribute in the arterial remodeling and in the atherogenesis [29].

To our surprise, LDLC was not a good measure of the relationships with the biomarkers studied. Its correlation coefficients with biomarkers were practically insignificant. Therefore, we would suggest that future studies of MetS use HDLC and TG as more reliable measures for the lipids component.

Our study has also limitations. We studied only 10 biomarkers. Their sample size depended on the cost of the biochemical assays. We have previously reported that about 25% of the FHS Time 2 participants studied used anti-hyperlipidemics, and that these medications were a confounder when studying the MetS prevalence [56]. It is known that such treatments affect concentrations in plasma of a few biomarkers studied. However, at present no medication summaries for biomarkers were available and thus, unfeasible to account for those effects.

In conclusions, PAI1 significantly correlated with most of the MetS conventional variables. PAI1 clustered in obesity and lipids factors, and participants in the PAI1 top 75% percentile had higher odds, than other 9 biomarkers, to be classified with MetS. PAI1 is an important risk factor for MetS. Three other biomarkers, CRP, IL6, and fibrinogen associate importantly with MetS cluster. In contrast DDIMER, SSA, and MMP3 did not associate directly with MetS cluster.

## Authors' contributions

All authors contributed equally.

## Appendix

### Hexagonal binning in our MetS study: Why it appeals as a reductionist technique

To the best of our knowledge, hexagonal binning is a technique that is not implemented in the epidemiological studies including MetS studies. It is applied in other areas of research, for example, earthquake graphical pattern detections [28]. Its main strength is in dealing with large datasets to clarify their spatial structure. The hexagonal binning technique was applied on the original data by creating larger units (16 bin counts per biomarker relative to its sample size) to reduce the dimensionality. Hexagon sizes (shown on the side of the graphs) within the Figures 1 and 2 represent the relative frequencies of subjects with similar biomarker values. This method was used to clarify the spatial structure of biomarkers against 0 to 5 categories of the NCEP MetS, while maintaining a measure of data density. The 0th MetS NCEP category refers to the fact that none of the 5 NCEP MetS definition thresholds was passed in a subject. The 1st category represents that at least one of the 5 thresholds was passed; similarly, the 5th MetS NCEP category it means that five of the MetS NCEP definition thresholds were passed in a subject (see **Metabolic syndrome **paragraph in the **Methods **section). In the hexagonal binning graphs (see Figures 1 and 2), three horizontal dashed lines represent the 25% (gray color), 50% (green color), and the 75% (red color) quartiles of a biomarker distribution. A regression line of the original biomarker values on MetS categories (0–5), together with its corresponding equation placed at the lower position of each graph helped to better understand the trend of the relation between a biomarker and MetS categories. To facilitate graphical perception and comparison, the BMI graph in relation to MetS categories was added in both Figures 1 and 2. Observations from the graphs in each figure show: (a) the densest distribution of subjects with 3 or more categories beyond the NCEP MetS definition thresholds; (b) the trend of the frequencies of subjects with MetS change in relation to the increasing values of a biomarker. The regression line between NCEP MetS categories and a specific biomarker adds information to the trends detected. For example, in the case of DDIMER and MMP3, most of subjects classified with MetS were heavily concentrated within q_25 _and q_75 _quartiles values of each of these biomarkers, but the biomarker relation trend with categories of MetS remained flat. This reinforced our previous conclusions that with the studied sample it is less probable that DDIMER and MMP3 contributed in the development or were associated with MetS. In the BMI graph one can see that the frequency of subjects with MetS increased as the BMI values augmented. From the Figure 1 it is clear more than half of the subjects with BMI above 31.9 (q_75_) were classified with MetS. Although a small group of subjects were with BMI less than 25 (q_25_), they also were classified with MetS. This shows that MetS is not only result of obesity in combination with other components of MetS, but it can also be a hypertension-dyslipidemic-hyperglycemic combination. For the PAI1 biomarker, (Figure 1), subjects with PAI1 greater than 54.5 (q_75_) were predominantly classified with MetS. In conclusion, hexagonal binning was a spatial graphical attempt to better characterize and understand the contributions of each biomarker studied in relation to MetS NCEP categories.

## References

[B1] (2002). Third report of the National Cholesterol Education Program (NCEP) expert panel on detection, evaluation and treatment of high blood cholesterol in adults (Adult Treatment Panel III). Final report. Circulation.

[B2] Rosenson RS (2005). Assessing risk across the spectrum of patients with the metabolic syndrome. Am J Cardiol.

[B3] Moller DE, Kaufman KD (2005). Metabolic syndrome: a clinical and molecular perspective. Annu Rev Med.

[B4] Bassuk SS, Rifai N, Ridker PM (2004). High -sensitive C-reactive protein: clinical importance. Curr Probl Cardiol.

[B5] Libby P, Ridker PM, Maseri A (2002). Inflammation and atherosclerosis. Circulation.

[B6] Stenvinkel P (2001). Endothelial dysfunction and inflammation – is there a link?. Nephrol Dial Transplant.

[B7] Mertens I, Verrijken A, Michiels JJ, Van der Planken M, Ruige JB, Van Gaal LF (2006). Among inflammation and coagulation markers, PAI-1 is a true component of the metabolic syndrome. Int J Obes (Lond).

[B8] Arya R, Blangero J, Williams K, Almasy L, Dyer TD, Leach RJ, O'Connell P, Stern MP, Duggirala R (2002). Factors of insulin resistance syndrome-related phenotypes are linked to genetic locations on chromosomes 6 and 7 in nondiabetic mexican-americans. Diabetes.

[B9] Meigs JB (2000). Insulin resistance syndrome? Syndrome X? Mulitple metabolic syndrome? A syndrome at all? Factor analysis reveals patterns in the fabric of correlated metabolic risk factors. Am J Epidemiol.

[B10] Yudkin JS, Juhan-Vague I, Hawe E, Humphries SE, di Minno G, Margaglione M, Tremoli E, Kooistra T, Morange PE, Lundman P, Mohamed-Ali V, Hamsten A, The HIFMECH Study Group (2004). Low-grade inflammation may play a role in the etiology of the metabolic syndrome in patients with coronary heart disease: the HIFMECH study. Metabolism.

[B11] Kraja AT, Rao DC, Weder AB, Mosley TH, Turner ST, Hsiung CA, Quertermous T, Cooper R, Curb JD, Province MA (2005). An Evaluation of the Metabolic Syndrome in a Large Multi-Ethnic Study: the Family Blood Pressure Program. Nutr Metab (Lond).

[B12] Sakkinen PA, Wahl P, Cushman M, Lewis MR, Tracy RP (2000). Clustering of Procoagulation, Inflammation, and Fibrinolysis Variables with Metabolic Factors in Insulin Resistance Syndrome. Am J Epidemiol.

[B13] Tang W, Miller MB, Rich SS, North KE, Pankow JS, Borecki IB, Myers RH, Hopkins PN, Leppert M, Arnett DK (2003). Linkage analysis of a composite factor for the multiple metabolic syndrome: the National Heart, Lung, and Blood Institute Family Heart Study. Diabetes.

[B14] Hanley AJ, Festa A, D'Agostino RB, Wagenknecht LE, Savage PJ, Tracy RP, Saad MF, Haffner SM (2004). Metabolic and inflammation variable clusters and prediction of type 2 diabetes: factor analysis using directly measured insulin sensitivity. Diabetes.

[B15] Laaksonen DE, Niskanen L, Nyyssonen K, Punnonen K, Tuomainen TP, Salonen JT (2005). C-reactive protein in the prediction of cardiovascular and overall mortality in middle-aged men: a population-based cohort study. Eur Heart J.

[B16] Higgins M, Province M, Heiss G, Eckfeldt J, Ellison RC, Folsom AR, Rao DC, Sprafka JM, Williams R (1996). NHLBI Family Heart Study: objectives and design. Am J Epidemiol.

[B17] Ellison RC, Zhang Y, Wagenknecht LE, Eckfeldt JH, Hopkins PN, Pankow JS, Djousse L, Carr JJ (2005). Relation of the metabolic syndrome to calcified atherosclerotic plaque in the coronary arteries and aorta. Am J Cardiol.

[B18] Lukaski HC, Livingston GE (1989). Use of bioelectric impedance analysis to assess human body composition: a review. Nutritional status assessment of the individual.

[B19] Boneu B, Aptel I, Nguyen F, Canbus JP, Thirion C, Amiral J, Boccalon H, Elias A (1997). Liatest D-Di, a new fast assay to determine D-Dimers, has performances comparable to classical ELISA for diagnosis of deep vein thrombosis. Thromb Haemostasis.

[B20] Clauss A (1957). Gerrinnungsphysiologische schnellmethode zur Bestimmung des Fibrinogens. Acta Haematol.

[B21] Blum A, Sclarovsky S, Rehavia E, Shohat B (1994). Levels of T-lymphocyte subpopulations, interleukin-1β, and soluble interleukin-2 receptor in acute myocardial infarction. Am Heart J.

[B22] Harris TB, Ferrucci L, Tracy RP, Corti MC, Wachholder S, Ettinger WH, Heimovitz H, Cohen HJ, Wallace R (1999). Associations of elevated interleukin-6 and C-reactive protein levels with mortality in the elderly. Am J Med.

[B23] DeClerck P, Alessi M, Verstreken M, Kruithof E, Juhan-Vague I, Collen D (1988). Measurement of plasminogen activator inhibitor 1 (PAI-1) in biological fluids with a murine monoclonal antibody based enzyme-linked immunosorbent assay. Blood.

[B24] Macy EM, Meilahn EN, Declerck PJ, Tracy RP (1993). Sample preparation for plasma measurement of plasminogen activator inhibitor-1 antigen in large population studies. Arch Pathol Lab Med.

[B25] Tracy R, Bovill E, Beutler E, Lichtman M, Coller B, Kipps T (1995). Plasminogen activator inhibitor-1. Williams Hematology.

[B26] Ridker PM, Hennekens CH, Roitman-Johnson B, Stampfer MJ, Allen J (1998). Plasma concentration of soluble intercellular adhesion molecule 1 and risks of future myocardial infarction in apparently healthy men. Lancet.

[B27] Labarrere CA, Nelson DR, Faulk WP (1997). Endothelial activation and development of coronary artery disease in transplanted human hearts. JAMA.

[B28] Carr DB, Olsen AT, White D (1992). Hexagon mosaic maps for display of univariate and bivariate geographical data. Cartography and Geographical Information Systems.

[B29] Libby P, Theroux P (2005). Pathophysiology of coronary artery disease. Circulation.

[B30] Aso Y, Wakabayashi S, Yamamoto R, Matsutomo R, Takebayashi K, Inukai T (2005). Metabolic syndrome accompanied by hypercholesterolemia is strongly associated with proinflammatory state and impairment of fibrinolysis in patients with type 2 diabetes: synergistic effects of plasminogen activator inhibitor-1 and thrombin-activatable fibrinolysis inhibitor. Diabetes Care.

[B31] Rudin E, Barzilai N (2005). Inflammatory peptides derived from adipose tissue. Immunity & Ageing.

[B32] Gimeno RE, Klaman LD (2005). Adipose tissue as an active endocrine organ: recent advances. Curr Opin Pharmacol.

[B33] Liang X, Kanjanabuch T, Mao S, Hao CM, Tang TW, Declerck PJ, Hasty AH, Wasserman DH, Fogo AB, Ma LJ (2006). Plasminogen activator inhibitor-1 modulates adipocyte differentiation. Am J Physiol Endocrinol Metab.

[B34] Kershaw EE, Flier JS (2004). Adipose tissue as an endocrine organ. J Clin Endocrinol Metab.

[B35] Fay WP, Parker AC, Condrey LR, Shapiro AD (1997). Human plasminogen activator inhibitor-1 (PAI-1) deficiency: characterization of a large kindred with a null mutation in the PAI-1 gene. Blood.

[B36] Swiatkowska M, Szemraj J, Cierniewski CS (2005). Induction of PAI-1 expression by tumor necrosis factor alpha in endothelial cells is mediated by its responsive element located in the 4G/5G site. FEBS J.

[B37] Vaughan DE (2005). PAI-1 and atherothrombosis. J Thromb Haemost.

[B38] Erickson LA, Fici GJ, Lund JE, Boyle TP, Polites HG, Marotti KR (1990). Development of venous occlusions in mice transgenic for the plasminogen activator inhibitor-1 gene. Nature.

[B39] Eren M, Painter CA, Atkinson JB, Declerck PJ, Vaughan DE (2002). Age-dependent spontaneous coronary arterial thrombosis in transgenic mice that express a stable form of human plasminogen activator inhibitor-1. Circulation.

[B40] Smith A, Patterson C, Yarnell J, Rumley A, Ben-Shlomo Y, Lowe G (2005). Which hemostatic markers add to the predictive value of conventional risk factors for coronary heart disease and ischemic stroke? The Caerphilly Study. Circulation.

[B41] Trayhurn P, Wood IS (2005). Signalling role of adipose tissue: adipokines and inflammation in obesity. Biochem Soc Trans.

[B42] Skurk T, Hauner H (2004). Obesity and impaired fibrinolysis: role of adipose production of plasminogen activator inhibitor-1. Int J Obes Relat Metab Disord.

[B43] Ridker PM, Wilson PW, Grundy SM (2004). Should C-reactive protein be added to metabolic syndrome and to assessment of global cardiovascular risk?. Circulation.

[B44] Biasucci LM, Giubilato G, Graziani F, Piro M (2005). CRP is or is not a reliable marker of ischaemic heart disease?. Lupus.

[B45] Lloyd-Jones DM, Greenland P (2004). Letter regarding article by Ridker et al, "Should C-reactive protein be added to metabolic syndrome and to assessment of global cardiovascular risk?". Circulation.

[B46] Pankow JS, Folsom AR, Cushman M, Borecki IB, Hopkins PN, Eckfeldt JH, Tracy RP (2001). Familial and genetic determinants of systemic markers of inflammation: the NHLBI family heart study. Atherosclerosis.

[B47] Pedersen BK, Febbraio M (2005). Muscle-derived interleukin-6-a possible link between skeletal muscle, adipose tissue, liver, and brain. Brain Behav Immun.

[B48] Wallenius K, Jansson JO, Wallenius V (2003). The therapeutic potential of interleukin-6 in treating obesity. Expert Opin Biol Ther.

[B49] Di Gregorio GB, Hensley L, Lu T, Ranganathan G, Kern PA (2004). Lipid and carbohydrate metabolism in mice with a targeted mutation in the IL-6 gene: absence of development of age-related obesity. Am J Physiol Endocrinol Metab.

[B50] Fasshauer M, Paschke R, Stumvoll M (2004). Adiponectin, obesity, and cardiovascular disease. Biochimie.

[B51] Kralisch S, Klein J, Lossner U, Bluher M, Paschke R, Stumvoll M, Fasshauer M (2005). Interleukin-6 is a negative regulator of visfatin gene expression in 3T3-L1 adipocytes. Am J Physiol Endocrinol Metab.

[B52] Ridker PM, Rifai N, Pfeffer M, Sacks F, Lepage S, Braunwald E (2000). Elevation of tumor necrosis factor-alpha and increased risk of recurrent coronary events after myocardial infarction. Circulation.

[B53] Huang Miao-Tzu, Mason JustinC, Birdsey GraemeM, Amsellem Valerie, Gerwin Nicole, Haskard DorianO, Ridley AnneJ, Randi AnnaM (2005). Endothelial intercellular adhesion molecule (ICAM)-2 regulates angiogenesis. Blood.

[B54] Duncan BB, Schmidt MI (2001). Chronic activation of the innate immune system may underlie the metabolic syndrome. Sao Paulo Med J.

[B55] Forsythe CE, Phinney SD, Fernandez ML, Quann EE, Wood RJ, Bibus DM, Kraemer WJ, Feinman RD, Volek JS (2008). Comparison of low fat and low carbohydrate diets on circulating fatty acid composition and markers of inflammation. Lipids.

[B56] Kraja AT, Borecki IB, North K, Tang W, Myers RH, Hopkins PN, Arnett D, Corbett J, Adelman A, Province MA (2006). Longitudinal and age trends of metabolic syndrome and its risk factors: The Family Heart Study. Nutr Metab (Lond).

